# Water-Soluble Star Polymer as a Potential Photoactivated Nanotool for Lysozyme Degradation

**DOI:** 10.3390/polym16020301

**Published:** 2024-01-22

**Authors:** Lidia Mezzina, Angelo Nicosia, Laura Barone, Fabiana Vento, Placido Giuseppe Mineo

**Affiliations:** 1 Department of Chemical Sciences and INSTM UdR of Catania, University of Catania, V.le A. Doria 6, I-95125 Catania, Italy; lidia.mezzina@phd.unict.it (L.M.); barone.laura@studium.unict.it (L.B.); fabianav2@libero.it (F.V.); 2 Institute for Chemical and Physical Processes, National Research Council (IPCF-CNR), Viale F. Stagno d’Alcontres 37, I-98158 Messina, Italy; 3 Institute of Polymers, Composites and Biomaterials, National Research Council (IPCB-CNR), Via P. Gaifami 18, I-95126 Catania, Italy

**Keywords:** porphyrin, protein degradation, photo-degradation, lysozyme, l-tryptophan

## Abstract

The development of nanotools for chemical sensing and macromolecular modifications is a new challenge in the biomedical field, with emphasis on artificial peptidases designed to cleave peptide bonds at specific sites. In this landscape, metal porphyrins are attractive due to their ability to form stable complexes with amino acids and to generate reactive oxygen species when irradiated by light of appropriate wavelengths. The issues of hydrophobic behavior and aggregation in aqueous environments of porphyrins can be solved by using its PEGylated derivatives. This work proposes the design of an artificial photo-protease agent based on a PEGylated mercury porphyrin, able to form a stable complex with l-Tryptophan, an amino acid present also in the lysozyme structure (a well-known protein model). The sensing and photodegradation features of PEGylated mercury porphyrin were exploited to detect and degrade both l-Trp and lysozyme using ROS, generated under green (532 nm) and red (650 nm) light lasers. The obtained system (Star3600_Hg) and its behavior as a photo-protease agent were studied by means of several spectroscopies (UV-Vis, fluorescence and circular dichroism), and MALDI-TOF mass spectrometry, showing the cleavage of lysozyme and the appearance of several short-chain residues. The approach of this study paves the way for potential applications in theranostics and targeted bio-medical therapies.

## 1. Introduction

One of the challenges in the biomedical field is to study new systems that act as nano-tools for chemical sensing or modifications at molecular levels, minimizing immune response or biodegradation problems [1].

More focus has been placed on the creation of site-specific protein-cleaving agents, which is a remarkable challenge due to the exceptional stability of peptide bonds [2]. One of the advances in the use of artificial peptidases lies in the possibility of mapping and discerning binding sites on proteins and in the possibility of using them as molecular scissors to generate smaller fragments suitable for protein sequencing studies [3], the design of new DNA-targeted drugs [4] or even for the production of new biomaterials [5].

A commonly used approach is to design specific chemicals capable of binding to proteins at specific sites and cleaving their backbone when exposed to light irradiation [6]. This purpose could be pursued by using specific classes of chromophores that, in the presence of specific molecules, form complexes and modify their physicochemical properties as a result of structural modifications or binding processes [6]. Among these systems, porphyrins draw great attention because of the possibility of coordinating a metal in the center of the tetra pyrrolic core, which can then interact with several kinds of biologically interesting molecules such as amino acids [7,8].

In recent years, porphyrins have been widely used in sensing in solid state [9,10], in solution [11,12], and in theranostics (a field of medicine that combines therapy and diagnostics) due to their unique properties and versatility [13,14,15]. In particular, porphyrins are among the best candidates for non-invasive diagnostics (such as imaging) since they can be used as contrast agents in techniques like magnetic resonance imaging (MRI), positron emission tomography (PET) and fluorescence imaging [16,17,18].

Furthermore, porphyrins are particularly suitable to be employed in targeted therapies, such as photodynamic therapy (PDT) [19]. This exploits the synergistic effect of a photosensitizer that, when irradiated with a proper light source, can react with oxygen in its fundamental state to initiate a well-known photochemical process that leads to the generation of reactive oxygen species (ROS) such as singlet oxygen, ^1^O_2_ [20], a cytotoxic agent [15]. However, in PDT, the appropriate wavelength of the excitation source is important. In fact, a light range between 500 and 900 nm is needed, since this is the light range where tissues are more optically transparent. Moreover, the use of high-energy ultraviolet (UV) and near-UV wavelengths can lead to DNA and cellular damage [21].

Even if porphyrins are excellent candidates for PDT, they suffer from hydrophobic behavior due to their highly conjugated structure and hence the tendency to aggregate in aqueous media, such as the cell environment [22]. To overcome this issue, a variety of approaches can be used [17], such as the loading of porphyrin onto nanoparticles [23], their incorporation into hydrogels [24] or coupling with cyclodextrins [25]. In this context, the use of ionic porphyrins (e.g., the meso-tetrakis(p-sulfonatophenyl)porphyrin) has many drawbacks, such as sensitivity to environmental conditions including the pH and ionic strength of a solution [26,27,28]. Moreover, the presence of charges in the porphyrin structure could determine the denaturation of proteins and/or hinder the penetration of the porphyrin itself inside cells, aside from the non-specific binding that can lead the porphyrin to be bound to proteins or biomolecules with complementary charges rather than the target protein [29]. An alternative viable option is the use of PEGylated porphyrins [30], where the presence of PEG chains in peripheral positions with an appropriate polymerization degree [30,31] renders the porphyrin suitable for biomedical applications (avoiding the aggregation phenomena), guaranteeing excellent water solubility, stealth behaviors towards the immune system and biocompatibility [30].

Lysozyme is one of the most studied proteins, since it has several valuable properties and is widespread [32]. Moreover, it shows bacteriostatic and bactericidal properties [33] and could be used as a food preservative to prevent the growth of food spoilage microorganisms, carrying out its antibacterial activity by acting as a catalyst for the hydrolysis of specific polysaccharides contained in bacteria’s cell walls [34].

In this work, we designed an artificial photo-protease agent able to form a complex with l-Tryptophan (l-Trp) residue that, when irradiated by light with a suitable wavelength, determines the amino acid/protein degradation. In particular, several metal porphyrin derivatives were investigated as l-Trp sensors and photosensitizers.

Taking into account the sensing and the photochemical features of a PEGylated porphyrin with a mercury atom complexed into the tetrapyrrolic core (Star3600_Hg), and the capability of l-Trp to act as an intermediate in the photo-oxidation mechanism involving the production of singlet oxygen, the above features were exploited to determine the selective degradation of lysozyme polypeptide. The synthesized systems and photodegradation features were investigated by means of MALDI-TOF mass spectrometry and UV-Vis, fluorescence and circular dichroism spectroscopies. To the best of our knowledge, this is the first case reported about a water-soluble mercury porphyrin suitable to be used as an artificial protease.

## 2. Materials and Methods

Metal acetates, chloroform, ethanol, triethylamine, LC-MS-grade water and pyridine were purchased from Sigma Aldrich (Merck Group, Milan, Italy).

### 2.1. Synthesis of 5,10,15,20-Tetrakis-p-(ω-methoxy-poly-oxyethylene phenyl) Porphyrin (Star3600_2H)

Briefly, a solution of 1.57 g of PEGMEC (2.15 mmol) solubilized in 9 mL of H_2_O:THF solution (1:1) was added to 0.18 g of P4OH (0.27 mmol) previously solubilized in 2.2 mL of an aqueous solution of NaOH 0.5 M, and stirred for 24 h. Then, a further 2.2 mL of NaOH 0.5 M and 2 mL of THF were added to the mixture and allowed to react for 24 h more. The reaction product was acidified and dried under a nitrogen stream and then in a vacuum oven (80 °C for 48 h). The residue was solubilized in CHCl_3_ and purified via chromatography using silica gel as the stationary phase and the mixture CHCl_3_:EtOH:N(C_2_H_5_)_3_ (96.5:2.0:1.5) as the eluent, resulting in a yield of 30%.

### 2.2. Synthesis of the 5,10,15,20-Tetrakis-p-(ω-methoxy-poly-oxyethylene phenyl) Metal-Porphyrins (Star3600_Me)

The synthesis of metal porphyrins involved the reaction between Star3600_2H and the acetate of the chosen metal. Briefly, 100 µmol of the desired metal salt (acetate of nickel, zinc, tin, manganese, rhodium, copper, erbium, mercury, iron) and 10 µmol of Star3600_2H (about 36 mg) were solubilized in 5 mL of pyridine. Subsequently, the reaction took place at 100 °C under continuous stirring for 4 h in a nitrogen atmosphere. Following this, the solvent was evaporated via a nitrogen stream, and the resulting residue was dried in a vacuum oven at 80 °C for two days. The selected 5,10,15,20-tetrakis-p-(ω-methoxy-polyethyleneoxy phenyl)-metal porphyrin, where the metal replaces the two hydrogen atoms within the tetrapyrrolic core, was isolated through column chromatography, employing silica gel as the stationary phase and a CHCl_3_:C_2_H_5_OH:N(C_2_H_5_)_3_ (96.5:2.0: 1.5) as the eluent. The first colored band was collected and corresponded to the metal porphyrin derivative, resulting in a yield of 85%.

### 2.3. Estimation of Singlet Oxygen Production

To quantitatively investigate the production of singlet oxygen, the standard method based on the *p*-nitroso-N,N’-dimethylaniline (RNO) bleaching reaction was used [35].

Briefly, the potential sensitizer (Star3600_Me, 5 µM), RNO (50 µM) and imidazole (10 mM) were dissolved in water. As the standard reference, a solution of 5,10,15,20-tetrakis(4-sulfonatophenyl)porphyrin (TPPS, 5 µM) was used. Photoactivation of the sensitizers was performed by irradiating the solution with a green (50 mW, λ = 532 nm) or red (100 mW, λ = 650 nm) laser source.

The quantum yield of ^1^O_2_ was calculated with the following relation [35]:

$$\Phi ={\;\Phi }_{TPPS}\frac{{OD}^{TPPS}}{{OD}^{Star3600\_Me}}\times \frac{{\Delta A}^{Star3600\_Me}}{{\Delta A}^{TPPS}}$$

where Φ_TPPS_ = ^1^O_2_ quantum yield of TPPS @532 nm, using imidazole as the acceptor [36]; OD is the optical density considering the source irradiation wavelength; and ΔA is the absorbance variation at 440 nm. OD and ΔA are related to both the standard and molecular probe.

### 2.4. Photodegradation Experiments

The photodegradation experiments were performed by using solid-state lasers (green, λ = 532 nm; red, λ = 650 nm) at a fixed distance from the quartz cuvette containing the appropriate solution target, which was kept under continuous stirring for the entire duration of the experiment. The photodegradation was monitored by acquiring UV-Vis, fluorescence and circular dichroism spectra of the solutions. The starting concentration was always 5 µM for Star3600_Hg, and the used ratios were 1:100 for the experiments involving l-Trp and 1:1 for the ones with LSZ.

### 2.5. Instruments

A Voyager DE (PerSeptive Biosystem, Waltham, MA, USA) equipped with a delay extraction device (operating at 25 kV applied voltage after 2600 ns delay, potential gradient of 454 V mm^−1^ and a wire voltage of 25 V) was used to acquire MALDI-TOF mass spectra, detecting positive ions in linear mode. The MALDI-TOF spectra of samples containing lysozyme were acquired in linear mode without the use of the delay extraction device. The matrices for the sample preparation were trans-2-[3-(4-tert-butylphenyl)-2-methyl-2-propenylidene] malononitrile (DCTB) for Star3600_2H and Star3600_Me, and a sum of 2,5-di hydroxybenzoic acid and 2-hydroxy-5-methoxybenzoic acid (SuperDHB) in 1:2 acetonitrile/0.1% aqueous TFA [37]. The mass spectrometer was calibrated with a previously reported method [38]. Grams/386 software (Version 3.04, Galactic Industries Corp, Salem, NH, USA) has been used to determine the molecular weights [38]. The *m*/*z* value is attributed to the molecular ion, considering the most abundant isotope of each element in the molecule.

UV-Vis spectra were recorded with a Cary60 UV-Vis spectrophotometer (Agilent Technologies, Santa Clara, CA, USA), using quartz cuvettes (1 cm path length) and water as the solvent (T = 25.0 ± 0.1 °C).

Fluorescence spectra were acquired with a FP-8200 spectrofluorimeter (Jasco Corporation, Tokyo, Japan), using a quartz cuvette (1 cm path length) and water as a solvent (T = 25.0 ± 0.1 °C).

Absorbance and fluorescence spectra were processed with Spectragryph optical spectroscopy software (version 1.2.11, Dr. F. Menges, Oberstdorf, Germany, 2019) [39].

The CD spectra were acquired using a JASCO J-815 spectropolarimeter (Jasco Corporation, Tokyo, Japan) with a 150 W Xenon lamp. The ellipticity, θ ∝ ε_L_–ε_R_, was obtained by calibrating the instrument with a 0.06% aqueous solution of [D_10_] camphorsulfonate. The measurements were performed with a quartz cuvette (0.2 and 0.5 cm path length) at constant temperature (T = (20.0 ± 0.1 °C)), using a Peltier thermostat device (JASCO PTC-423S/15, Jasco Corporation, Tokyo, Japan).

## 3. Results and Discussion

### 3.1. Structural and Spectroscopic Characterization of Star3600_2H and Star3600_Me

Star3600_2H has been synthesized through the etherification reaction between the phenyl groups of 5,10,15-tetrakis (p-hydroxyphenyl) porphyrin and the poly(ethylene glycol) methyl ether chloride (PEGMEC_750_). To ensure the full solubility of the porphyrin in aqueous media, we have chosen to use PEG branches with an average polymerization degree (Xn) of about 16.

**Figure 1:**
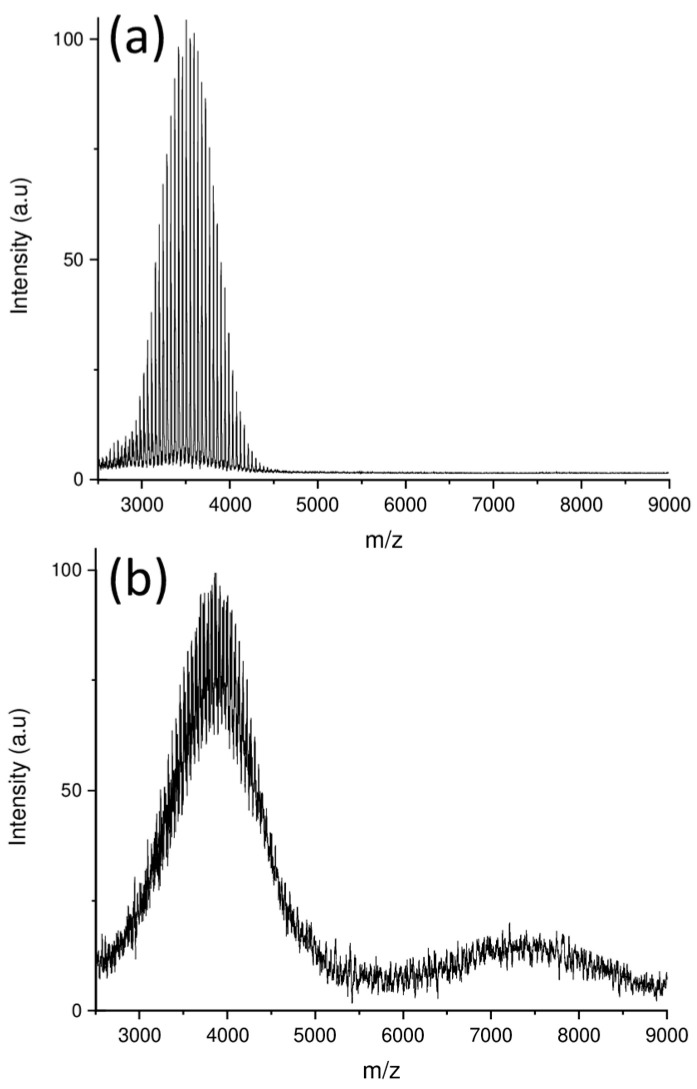
MALDI-TOF mass spectra of Star3600_2H (**a**) and Star3600_Hg (**b**).

The structure of the Star3600_2H was confirmed through MALDI-TOF mass spectrometry analysis (Figure 1). Specifically, the molar mass distribution, centered at *m*/*z* = 3504, has shown peaks that are detected at *m*/*z* values of 734 + n44, with n ranging from 43 to 79, attributable to MK^+^ adducts species.

**Scheme 1:**
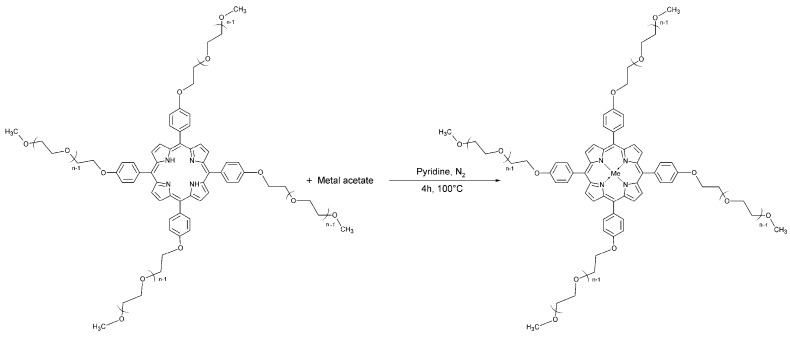
Exemplificative scheme of the synthesis of Star3600_Me.

The metalated derivatives were produced through a reaction involving Star3600_2H and the acetate salt of the selected metal in pyridine solution at 100 °C (see Scheme 1 and the experimental section). The confirmation of the metal insertion inside the porphyrin core was obtained via MALDI-TOF mass spectrometry and both UV-Vis and fluorescence spectroscopies. As an example, in Figure 1b the MALDI-TOF spectrum of Star3600_Hg is reported (mass spectra of each Star3600_Me derivative are not reported for brevity). It shows a series of peaks with a molecular mass distribution shifted to higher *m*/*z* values (about 200 uma) than that of the free-base counterpart (Figure 1a). Moreover, the presence of a broadened peak distribution is noticeable between *m*/*z* = 6000 and 9000, which can be attributed to the (Star3600-Hg)_2_ species having two Hg ions coordinated by two porphyrins [40].

**Figure 2:**
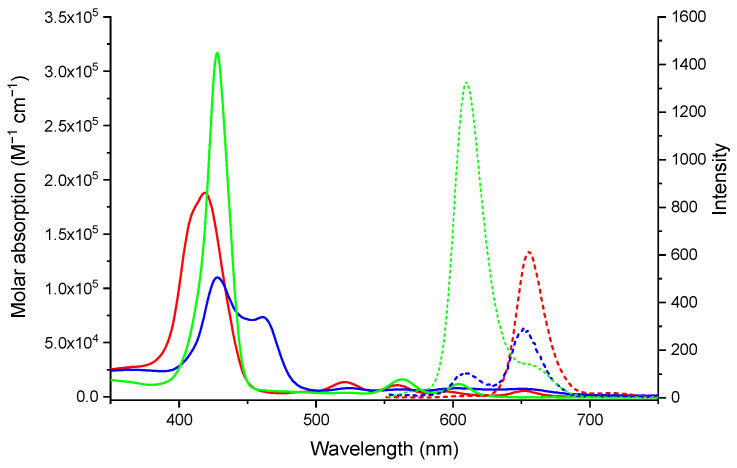
UV-Vis (continuous lines) and fluorescence (dashed lines) spectra of water solutions of Star3600_2H (red lines, λ_exc_ = 430 nm), Star3600_Zn (green lines, λ_exc_ = 435 nm) and Star3600_Hg (blue lines, λ_exc_ = 440 nm. Spectrum magnified ×25).

The UV-vis spectrum of Star3600_2H shows a Soret band at 419 nm and four Q-bands at 520, 559, 595 and 652 nm, respectively. As expected for metal porphyrins, Star3600_Zn (Figure 2, green line) shows a bathocromic shift of the Soret band of about 8 nm, which now appears at 428 nm, and the disappearance of two Q-bands, with the other two remaining at 563 and 603 nm (similar spectra are observed for all porphyrin–metal complexes, not reported for brevity). Typically, following Gouterman’s four orbitals model and due to the π → π* transitions between the HOMO and LUMO, the number of Q-bands arises from the combination of different electronic transitions involving the central metal ion and the porphyrin ligands [31]. In particular, when a metal ion is located between the pyrrolic nitrogens, due to an increase in the symmetry of the porphyrin itself, a decrease in the number of Q-bands occurs [41].

On the other hand, the UV-vis spectrum of Star3600_Hg shows a Soret band at about 428 nm overlapped with a band at 461 nm, and four Q-bands (respectively, at 524, 561, 601 and 654 nm). The presence of the doubled Soret band and of the four Q-bands suggest the presence of both the Star3600_Hg (porphyrin-Hg 1:1 ratio) and the (Star3600-Hg)_2_ systems (porphyrin-Hg 2:2 ratio), and it could be hypothesized that the Soret band at 428 nm is due to the 1:1 complex, while the one at 461 nm is attributable to the 2:2 complex. In this scenario, the observed UV-Vis spectrum derives from the overlapping of the spectra of both systems.

As concerns the fluorescence spectra (Figure 2, dashed lines), Star3600_2H exhibit a strong fluorescence at 655 nm, while the mercury atom into the porphyrin core causes a strong fluorescence quenching, showing a weak signal at 652 nm with a shoulder at 608 nm (for a clearer representation, the Star3600_Hg fluorescence spectrum in Figure 2 is magnified ×25). Instead, Star3600_Zn shows a higher fluorescence intensity with respect to the Star3600_2H and Star3600_Hg systems.

### 3.2. Star3600_Me Estimation of Singlet Oxygen Production

**Figure 3:**
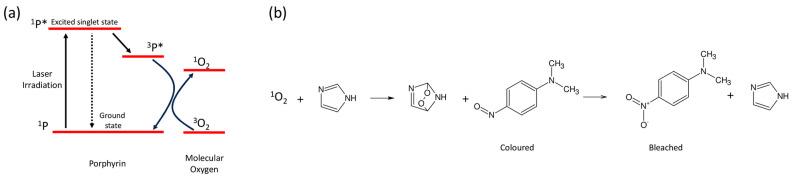
(**a**) Singlet oxygen production mechanism scheme and (**b**) RNO degradation mechanism mediated by the ^1^O_2_–imidazole system.

Porphyrins are widely used as photosensitizers thanks to their capability to generate reactive oxygen species when triggered by a suitable light source [42]. Indeed, once the photon is absorbed, determining an electron excitation from the ground state to the singlet state, the intersystem crossing process could generate an excited triplet state, in which energy might be transferred (in a non-radiative way) to the triplet ground state of the oxygen molecules (Figure 3a), generating singlet oxygen (^1^O_2_) species [43]. Usually, this last phenomenon is exploited in photodynamic therapy treatment.

To estimate the quantum yield in singlet oxygen production of the investigated systems under light laser triggering, a standard colorimetric method based on the RNO bleaching phenomenon was used. In this approach, the ^1^O_2_ species induce the RNO oxidation, exploiting the formation of a transient imidazole trans-annular peroxide intermediate (Figure 3b) [35].

Among the tested Star3600_Me systems (Me = nickel, zinc, tin, manganese, rhodium, copper, erbium, mercury, iron), only those containing Zn, Sn, Hg, Rh and Er ions can generate singlet oxygen species (the respective quantum yields are reported in Table 1). The inability of some metal to produce singlet oxygen (nickel, tin, manganese, copper, erbium, iron) porphyrins could be due to the static quenching phenomenon [30], hindering the attainment of the excited state [44].

### 3.3. Star3600_Me Sensing Properties toward Tryptophan

**Figure 4:**
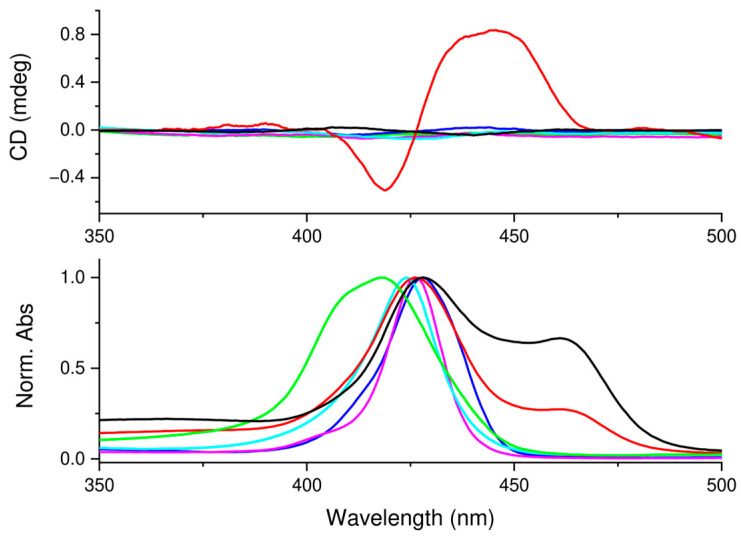
**Upper**: circular dichroism spectra of aqueous solutions of pristine Star3600_Hg (black line) and of Star3600_Er (green), Star3600_Rh (cyan), Star3600_Sn (magenta), Star3600_Zn (blue) and Star3600_Hg (red line) when mixed with l-Trp (molar ratio 1:100). **Bottom**: relative UV-Vis spectra in the Soret band region.

In addition to being used as photosensitizers for singlet oxygen production [45,46], some metal porphyrins are powerful macromolecular sensors for amino acids as well [47,48]. To verify the binding properties of the metal porphyrins (able to generate singlet oxygen) with respect to l-Trp, water solutions of Star3600_Me (4.6 × 10^−6^ M) were mixed with l-Trp in a molar ratio of 1:100, and UV-Vis and circular dichroism spectra were acquired. As expected, because the Er, Rh, Sn and Zn porphyrins do not form a stable chiral complex with l-Trp, silent circular dichroism spectra were recorded (Figure 4, green, cyan, magenta, blue lines, respectively).

On the other hand, the mixture of Star3600_Hg and l-Trp (Figure 4, red line) shows complex induced circular dichroism (ICD) signals in correspondence with the porphyrin Soret bands [49], due to the formation of a static complex. In particular, the experimental data suggest the formation of multiple kinds of chiral supramolecular systems, mainly due to the overlapping of negative–positive (centered at about 429 nm) and positive (centered at about 456 nm) ICD signals, in which l-Trp is coordinated by the metal of Hg porphyrins. In general, the induced chirality generating a bisignate signal arises when identical (or nearly identical) chromophores with strong extinction coefficients are found closely positioned in space, so they are able to display an observable interaction that stems from the efficient coupling of their electric transition moments [50].

**Figure 5:**
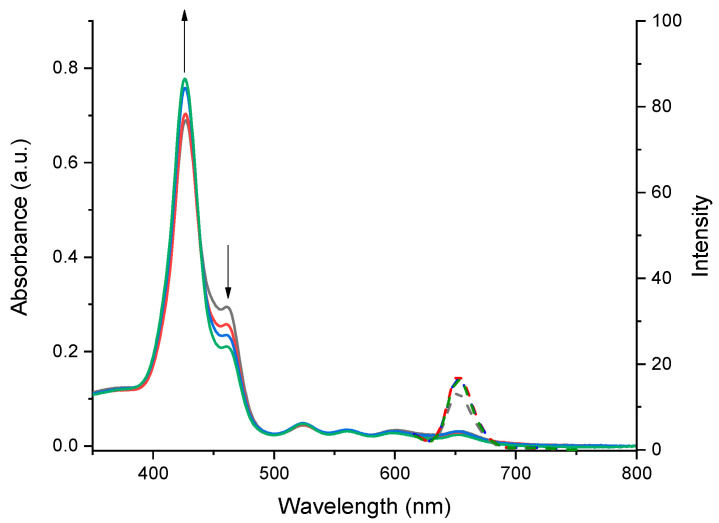
UV-vis (continuous lines) and fluorescence (dashed lines) spectra of aqueous solutions of Star3600_Hg (black line) and a mixture of Star3600_Hg and l-Trp in molar ratio 1:100 freshly prepared (red line) and after 10 and 120 min (blue and green lines, respectively).

To have a further understanding of the behavior of Star3600_Hg when interacting with l-Trp, the absorption and fluorescence features were investigated. As reported in Figure 5, due to the stabilization phenomenon between the two systems, there is a time-dependent interaction between l-Trp and Star3600_Hg, showing a simultaneous increase in the band at 426 nm and a decrease in the band at 461 nm. Considering the circular dichroism experimental results, this behavior could be explained since probably both Star3600_Hg and (Star3600-Hg)_2_ systems are able to create a stable complex with l-Trp [51]. Moreover, experimental data suggest that the (Star3600-Hg)_2_/l-Trp complex transmutes itself in the Star3600_Hg-/l-Trp system, decreasing its concentration. Regarding the fluorescence emission spectrum, it shows a small increase in the emission intensity.

### 3.4. Tryptophan Photo-Degradation

**Figure 6:**
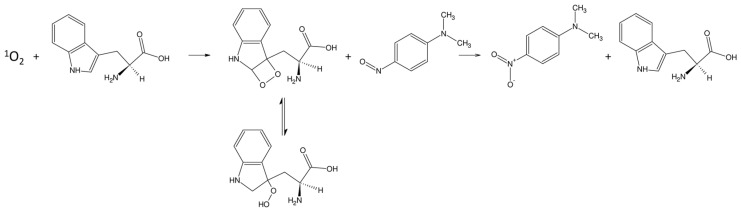
RNO degradation mechanism mediated by the ^1^O_2_–l-Trp system.

Considering that Star3600_Hg acts as an efficient sensor towards l-Trp creating a stable complex, and that l-Trp is potentially able to be an intermediate in the photo-oxidation mechanism, the features of both Star3600_Hg and l-Trp were investigated to determine the degradation of polypeptides containing l-Trp. In the supposed degradation mechanism, Star3600_Hg acts as a singlet oxygen source, while l-Trp serves both as a molecular target and as the singlet oxygen receptor. This interaction generates a peroxide species analogous to the mechanism observed with imidazole [52], capable of oxidizing the RNO system (Figure 6). Similarly, with the same mechanism, the system could be used to oxidize/damage the Trp-containing polypeptide. To validate this hypothesis, singlet oxygen detection experiments were conducted using l-Trp as the singlet oxygen intermediate receptor in place of imidazole.

The experiments were performed using TPPS, Star3600_2H (used as references) and Star3600_Hg photosensitizers. Moreover, the ability to generate singlet oxygen using the above systems was also investigated using a red light laser as a trigger. This wavelength range was selected because of the higher safety of this wavelength with respect to the green laser (less energetic), and because the light red region has a greater ability to penetrate the tissues.

For this purpose, a solution containing the sensitizer (5 µM), RNO (50 µM) and l-Trp (10 mM) was freshly prepared and then subjected to laser irradiation. Since the quantum yield of TPPS depends on the employed acceptor (l-Trp), in this case, we used Φ_TPPS-RED_ = 0.33 (^1^O_2_ quantum yield of TPPS@630 nm) and Φ_TPPS-GREEN_ = 0.50 (^1^O_2_ quantum yield of TPPS@540 nm) values [53]. The results are summarized in Table 2 and show that Star3600_Hg singlet oxygen production properties are effective under green and red laser irradiation.

**Figure 7:**
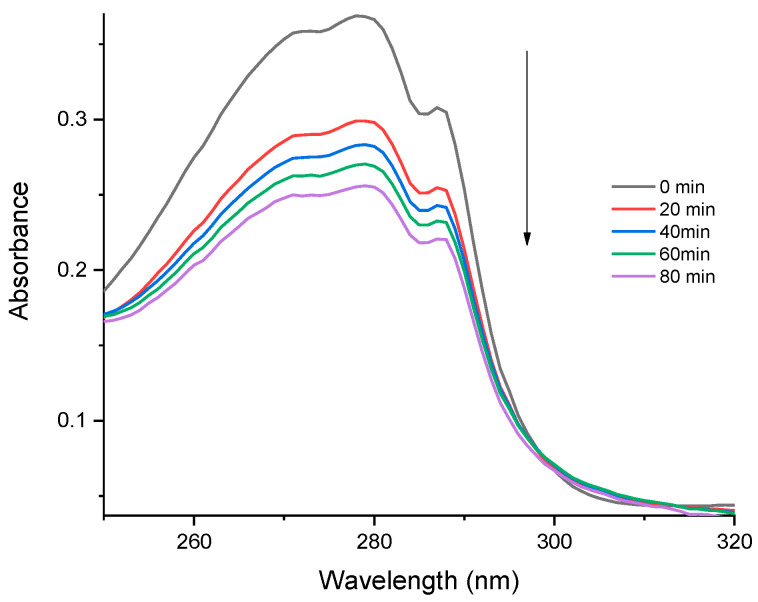
UV-vis spectra of the mixture of 5 µM aqueous solution of Star3600_Hg and l-TRP (ratio 1:100) when exposed to a red laser (650 nm) irradiation.

To shed light on the effect of Star3600_Hg acting as an artificial cleaving agent, l-Trp was used as a model since its residue can be found in many proteins. To verify that the formation of transannular species triggered by the porphyrinic photosensitizer induces the degradation of l-Trp, the disappearance of the peak at λ = 279 nm was monitored. Briefly, a 5 µM aqueous solution of Star3600_Hg was combined with l-Trp (ratio = 1:100) and irradiated with green or red laser light for a duration of 80 min. The change in its spectroscopic properties was observed by analyzing the same solution with absorbance spectroscopy (Figure 7), showing a strong decrease in the peak at λ = 279 nm (belonging to l-Trp). Similar results were obtained under green laser triggering. The experimental results confirm that, by exploiting this phenomenon, l-Trp can be a degradation site when located in protein chains.

### 3.5. Lysozyme Recognition and Degradation with Star3600_Hg

As a proof of concept of the feasibility of utilizing Star3600_Hg for the degradation of proteins containing l-Trp residues, the interaction of Star3600_Hg with lysozyme in aqueous solution (5 µM) was investigated. Lysozyme (LSZ) is a well-known protein, with an average molecular weight of about 14.6 kDa, and six tryptophane residues on its structure [54].

**Figure 8:**
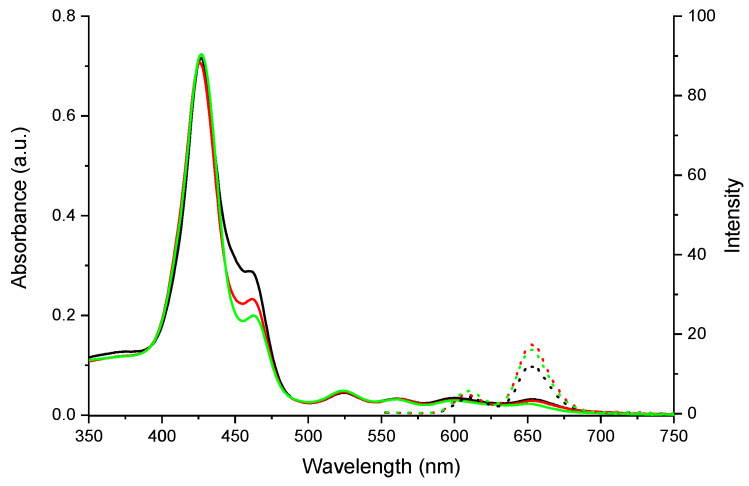
UV-Vis (continuous lines) and fluorescence (dashed lines) spectra (λ_exc_ = 440 nm) of Star3600_Hg/LSZ complex. The black line is an aqueous solution (5 µM) of Star3600_Hg, and red and green lines are the aqueous solution mixture of Star3600_Hg with LSZ (molar ratio 1:1) freshly prepared and after 2 days, respectively.

Specifically, the formation of the complex between Star3600_Hg and LSZ was investigated by means of absorbance and fluorescence spectroscopies. As shown in Figure 8, the formation of the complex leads to a decrease in the shoulder (λ = 461 nm) of the Star3600_Hg’s Soret band. Conversely, fluorescence spectra show an intensity increase upon the complex formation. These occurrences were similar to the one observed in the experiment involving l-Trp complexation, indicating the formation of the protein–porphyrin complex.

### 3.6. Light Irradiation Experiments

**Figure 9:**
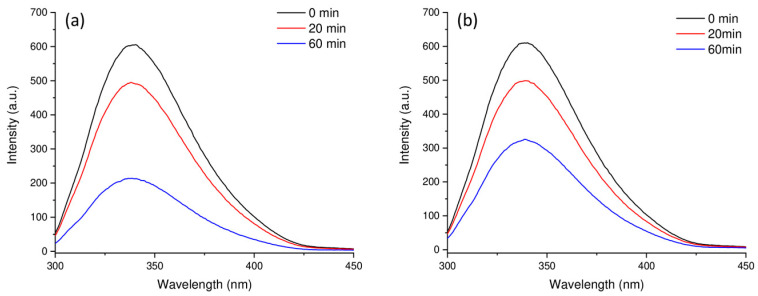
Fluorescence spectra (λ_exc_ = 290 nm) of the aqueous solution of Star3600_Hg and LSZ mixture (both 5 µM) under (**a**) green (532 nm) and (**b**) red (650 nm) laser irradiation.

In both the green (Figure 9a) and red (Figure 9b) laser light irradiation experiments, performed with a mixture of Star3600_Hg as the photosensitizer and LSZ as the target, the fluorescence spectra of LSZ (λ_exc_ = 290 nm) show an emission band at 338 nm that decreases its intensity during the irradiation time, suggesting a denaturation/degradation phenomenon in the polypeptide. In the absence of Star3600_Hg, any significant change in the LSZ emission band is recorded.

**Figure 10:**
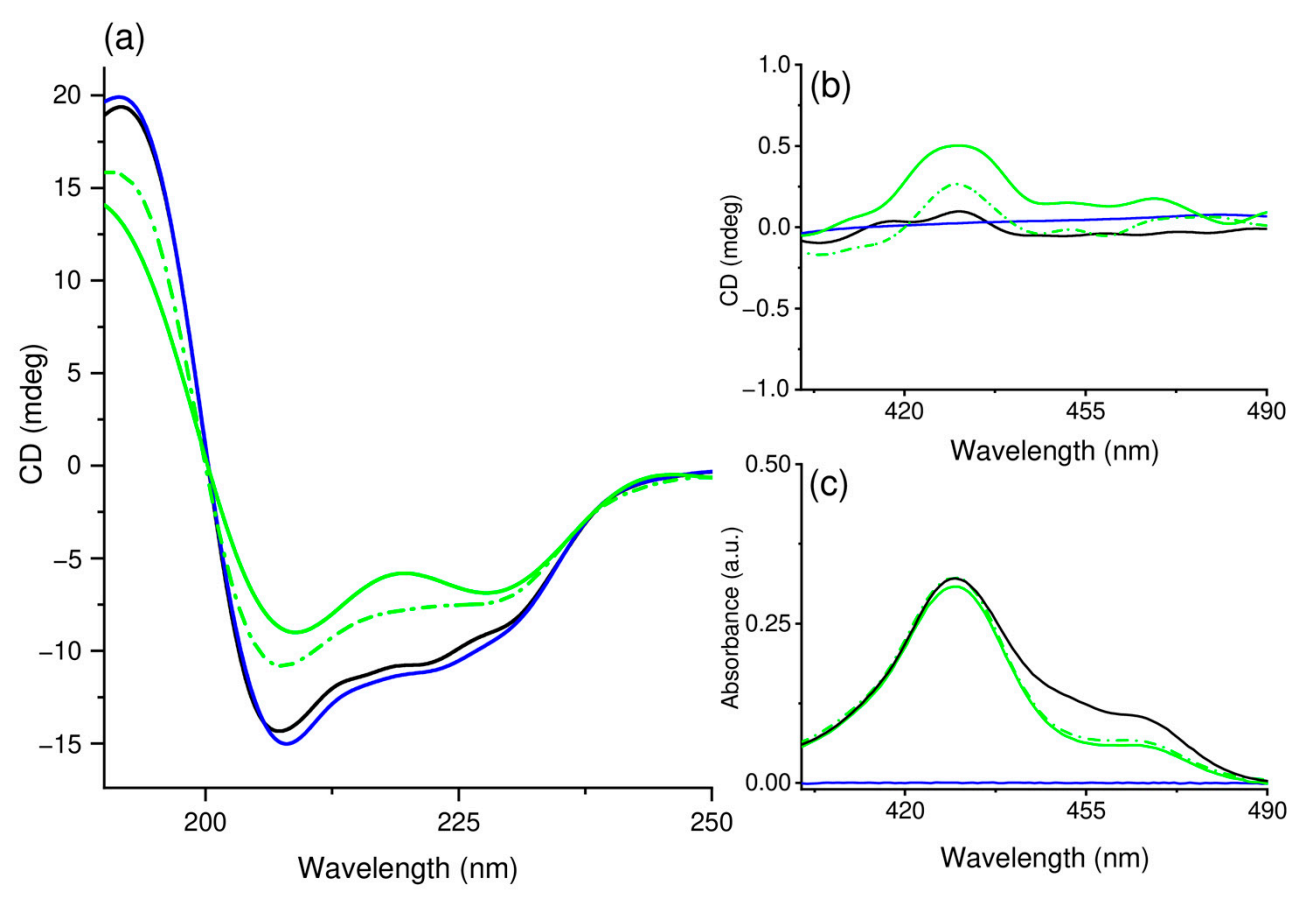
(**a**) CD spectra of aqueous solutions of LSZ (blue line), Star3600_Hg and LSZ (both 5 µM) mixture freshly prepared (black line), and after 40 (green dash dot line) and 80 (green solid line) minutes under green laser irradiation; (**b**) magnification of the range 400–490 nm and (**c**) the related UV-vis spectra.

Considering that far-UV CD spectra of proteins can give information about their secondary structure [55], specific circular dichroism experiments were performed before and after green laser irradiation (similar results were also obtained with red laser irradiation). The CD spectrum of native LSZ (Figure 10a, blue line) shows a positive band at 192 nm (attributable to the α-helical [56,57]) and two negative bands at 207 nm (π-π* transition) and 220 nm (n-π* transitions of both random coil and α-helical structures [58,59,60]). These bands overlap with those of the β-sheet structure, with a positive band at 195 nm and a negative one at 218 nm [57,61]. After irradiation with the green laser for 40 min (Figure 10a, dashed green line) and 80 min (Figure 10a, solid green line), a strong decrease in positive and negative bands and a modification in the CD shape were recorded. In particular, the CD spectrum of the solution irradiated for 80 min showed huge shape modifications in the far-UV range attributable to the occurrence of random coil structure arrangements [61]. Finally, after laser irradiation, a small ICD signal appears in the metal porphyrin Soret region (400–490 nm, Figure 10b), suggesting that the cleavage of the protein is occurring, setting oligopeptide sequences in solution. These latter can interact with the free Star3600_Hg system, leading to the formation of oligopeptide–Star3600_Hg chiral complexes. Supporting this interpretation, a correlation between the irradiation time and the ICD signal intensity is observed. On the other hand, the UV-Vis spectrum of the irradiated complex (Figure 10c) shows a decrease in the intensity of the (Star3600-Hg)_2_ system’s band (461 nm), as previous observed in the interaction between (Star3600-Hg)_2_ and l-Trp.

**Figure 11:**
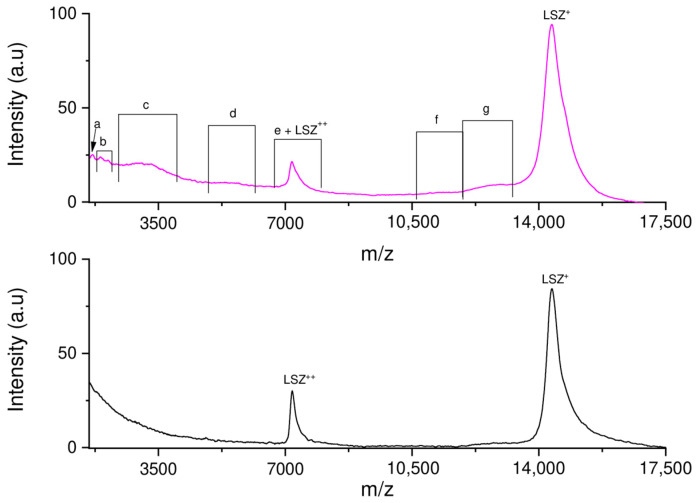
MALDI-TOF mass spectra of pristine lysozyme (black line) and Star3600_Hg-LSZ solution after 80 min of laser irradiation (magenta line). The symbols are explained in Table 3.

**Figure 12:**
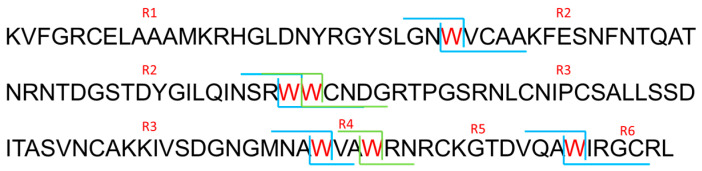
Aminoacidic sequence of lysozyme. W indicates l-Trp residues.

Finally, to have further confirmation of the scissor-like behavior of Star3600_Hg towards LSZ, the MALDI-TOF mass spectra of the pristine (Figure 11, black line) and 80 min irradiated (Figure 11, magenta line) Star3600_Hg-lysozime system were acquired. The MALDI-TOF spectrum of pristine lysozyme shows peaks due to the presence of the molecular species as single charge at about *m*/*z* 14400 (LSZ^+^) and double-charged species at about *m*/*z* 7200 (LSZ^++^). Instead, the irradiated mixture spectrum shows several broadened peaks, belonging to the different species originating from the cleavage of the sites where there are l-Trp residues, as shown in Table 3 and in Figure 12 (l-Trp residues are indicated with the red letter “W”). Based on the experimental data, we suppose that the aminoacidic sequence can be cleaved in every position (or neighboring to AA residues) where there are l-Trp residues. Similar results were obtained for both excitation wavelengths.

**Figure 13:**
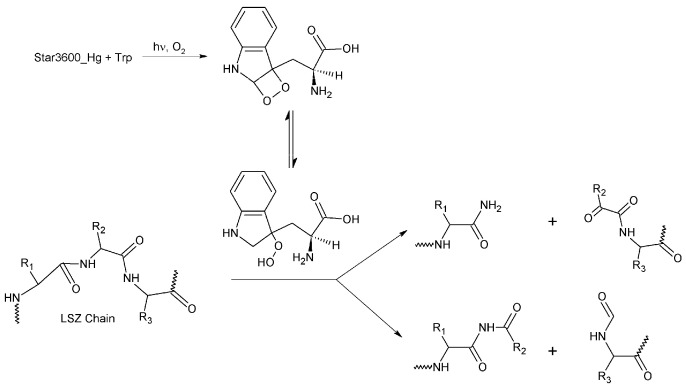
Schematic representation of the Star 3600_Hg photo-induced proteolysis mechanism.

Lysozyme degradation can be explained by considering two pathways: (i) Protein denaturation, which occurs following the light-triggering of Star3600_Hg with the formation of singlet oxygen and the following radical species. The latter can react with the disulfide bonds of proteins, determining the partial denaturation of lysozyme [62,63]. (ii) Cleavage of the polypeptide chain, due to the peroxide intermediate on the l-Trp residue and the consequent formation of radicals [64,65] that determine the l-Trp degradation and/or the polypeptide scissoring mechanism [52,66] (Figure 13).

## 4. Conclusions

In this work, we investigated the ROS-generating ability of several polymer-based metal porphyrin derivatives, and the potential use of l-Trp as a sensor and, when triggered by either red or green laser light, as a photocleaving agent.

The presence of hydrophilic poly(ethylene glycol) chains directly linked to the porphyrinic core allowed the full water solubility of Star3600_2H and of its metal complexes. Among the studied metal porphyrins, it was determined that only the Zn-, Sn-, Hg-, Rh- and Er-coordinated derivatives can generate singlet oxygen species.

Furthermore, within the investigated metal porphyrins, the coordination of mercury in the center of the porphyrinic core gives the porphyrin the ability to also act as a molecular sensor, leading to a stable complex with l-Trp. The phenomenon is easily recognizable from the changes in the spectroscopic properties of the porphyrin itself.

Since l-Trp resulted to be suitable as an acceptor agent of ^1^O_2_ species produced from the excitation of Star3600_Hg, and its residue can be found in several proteins, such as LSZ, the synergistic interaction of Star3600_Hg and l-Trp was exploited to selectively cleave the protein at sites where l-Trp residues are located. The fluorescence and circular dichroism spectroscopies and MALDI-TOF mass spectrometry investigations suggest the potential use of Star3600_Hg as a molecular scissor.

## Figures and Tables

**Table 1:** Quantum yields of the singlet oxygen under green laser irradiation (532 nm).

Photosensitizer	Quantum Yield
TPPS	0.62
Star3600_2H	0.34
Star3600_Zn	1.38
Star3600_Sn	0.94
Star3600_Hg	0.31
Star3600_Rh	0.31
Star3600_Er	0.25

**Table 2:** Quantum yields of the singlet oxygen under red (650 nm) and green laser irradiation (532 nm).

Photosensitizer	Φ_Red_	Φ_Green_
TPPS [53]	0.33	0.50
Star3600_2H	0.21	0.24
Star3600_Hg	0.22	0.60

**Table 3:** MALDI-TOF mass spectrum peaks attributions.

Label	Molecular Mass Range	Residue	Residue Amount (%)
a	1655	R5 + 2W	>1
b	1840–2170 Da	R4R5 + 2W, R4R5 + 3W, R5R6 + W	1
c	2560–3850 Da	R4R5R6 + 2W, R4R5R6 + 3W, R1, R1 + W, R2, R2 + W	7
d	4870–5480 Da	R3 + 2W, R3R4 + 2W, R3 + 3W, R3R4 + 3W, R3R4 + 4W	2
e	6860–7650 Da	R1R2 + W, R3R4R5 + 5W, R1R2 + 2W, R3R4R5R6 + 3W, R1R2 + 3W, R3R4R5R6 + 4W, R3R4R5R6 + 5W.Pristine lysozyme (double charge)	7
f	10,650–11,740 Da	R2R3R4R5 + 5W, R2R3R4R5 + 6W, R2R3R4R5R6 + 6W.	3
g	11,740–12,350 Da	R1R2R3 + 3W, R1R2R3 + 4W, R1R2R3R4 + 4W, R1R2R3R4R5 + 5W	10
LSZ	14,400 Da	Pristine lysozyme	69

## Data Availability

Data are contained within the article.
